# Predictors of vaping intention among adolescents: a systematic review

**DOI:** 10.1186/s12889-025-24518-x

**Published:** 2025-10-09

**Authors:** Muhammad Zulhilmie Saruddin, Rosliza Abdul Manaf, Khadijahtul Qubra Amizah Hamzah, Ainaa Athirah Ahmad Khusairi

**Affiliations:** 1 Department of Community Health, Faculty of Medicine and Health Sciences, Universiti Putra Malaysia, Serdang, 43400 Selangor Malaysia; 2 Department of Primary Care Medicine, Faculty of Medicine, University of Malaya, Kuala Lumpur, 50603 Malaysia

**Keywords:** Adolescent, E-cigarette, Vape, Intention, Susceptibility

## Abstract

**Background:**

Vaping behavior has risen sharply among adolescents, with intention to vape playing a significant role in initiating this practice. Adolescent experimentation with vapes remains a major public health concern. Identifying factors associated with vaping intention is essential for developing effective preventive measures before this risk behavior becomes established in adulthood. This systematic review aims to identify predictors of vaping intention among adolescents.

**Methods:**

The review follows the Preferred Reporting Items for Systematic Reviews and Meta-Analyses (PRISMA) 2020 statement. A comprehensive literature search was conducted across four databases (PubMed, CINAHL, Scopus and Science Direct), focusing on original English-language articles published between 2017 and 2022. Studies were screened and analyzed systematically using content analysis approach to identify key predictors.

**Results:**

In total, 13 studies were included. Several potential predictors of vaping intention were identified, including sociodemographic, personality and psychological, social, environment and tobacco cognitive factors.

**Conclusions:**

Effective prevention programs should address these modifiable risk factors through multifaceted interventions that simultaneously target multiple predictors. Registration: This systematic review is registered to PROSPERO Registry (reference number: CRD42024552998).

## Introduction

Adolescence is a period of significant developmental transition to adulthood [1]. According to the World Health Organization [2], adolescence can be defined as a phase of life between childhood and adulthood, from ages 10 to 19 years old. Adolescence is a crucial period during which long-term social and health behaviors are established. It is a critical time for adolescents to develop positive behavior patterns, healthy habits and independent decision-making skills that lay a strong foundation for a healthy lifestyle [1]. Cultivating these behaviors early may help prevent engagement in risk-taking activities, such as vaping, especially as behaviors established during adolescence often continue into adulthood. Since their introduction, vapes have dramatically altered the landscape of tobacco products, particularly among adolescents. This shift is evident in the recent decline in conventional cigarette use and the concurrent rise in vape use [3, 4].

Vaping among adolescents has emerged as global public health concern [5]. In the United States of America (US), the prevalence of past 30 days vaping among adolescents aged 14 to 18 years old has rapidly increased from 1.5% in 2011 to 20.8% in 2018 [6]. The 2023 National Youth Tobacco Survey (NYTS) reported that vape products were the most commonly used tobacco products among middle and high school students, with 2.13 million users (7.7%) [7]. A similar trend is observed in the United Kingdom (UK), where vaping prevalence among adolescents rose from 13.9% in 2020 to 20.5% in 2023 [8]. Vaping refers to the use of electronic cigarettes (EC) or other vaporizing devices, including cigarlike devices, tank systems, pods, personalize vaporizers, and similar products [9]. These handheld devices use an electrically powered coil to heat e-liquid solutions, converting them into an aerosol that is inhaled by the user [10]. Vaping poses serious health risks to adolescents, whose developing brains and bodies are particularly susceptible to harm. Early nicotine exposure can lead to addiction, disrupt brain development, and increase the risk of future substance use disorders [11]. The inhalation of vape aerosol has been associated with acute lung injury in cases involving illicit vaping products containing vitamin E acetate. While the long-term cardiovascular effects of vape use remain uncertain, ongoing research continues to evaluate potential health risks [12]. Moreover, reports of severe burns and ocular injuries due to device malfunctions highlight the physical dangers associated with vape use among young users [13, 14]. Understanding the factors influencing vaping intention, which defined as the absence of individual’s firm decision not to use vapes, is crucial to address this public health issue [15].

While numerous studies have examined the prevalence and health effects of adolescent vaping, a significant gap remains in understanding the specific factors driving vaping intention [16–18]. Existing literature has highlighted influences such as peer pressure, media exposure, and psychological traits [18, 19]. However, many of these studies primarily focused on external predictors, such as social and environmental factors, with less emphasis on personal factors such as behavioral intention, despite its central construct in understanding and predicting behavior [20, 21]. Vaping intention has been shown to be a strong predictor of both initiation and current use of vapes among adolescents. A longitudinal study in Texas reported that students with higher intention to vape had increased odds of both initiation (AOR = 2.46; 95% CI: 1.69–3.59; *p* < 0.001) and current use (AOR = 1.98; 95% CI: 1.07–3.69; *p* = 0.03) [22]. Similarly, Alduraywish et al. (2023) found that in Saudi Arabia, the intention to vape was a significant predictor of future use among adolescents (AOR = 3.5; 95% CI: 2.3–5.3; *p* < 0.001) [23].

The Theory of Planned Behavior (TPB) posits that intention is influenced by attitudes, subjective norms, and perceived behavioral control, making it a crucial determinant in shaping behavior [24]. Integrating TPB to the study of adolescent vaping provides a structured framework for understanding how personal traits, cognitions, and external influences interact to shape vaping intentions. Addressing behavioral intention through this theoretical lens not only bridges gaps in current research but also informs the development of targeted interventions to prevent vaping initiation among adolescents [21]. The aim of this systematic review is to synthesize existing evidence on the predictors of vaping intention among adolescents. Specifically, this review explores: [1] the psychological factors identified in previous research that contribute to vaping intention [2], the influence of social factors, including peer pressure and family attitudes, and [3] the role of advertising and media exposure in shaping perceptions and intention toward vaping. By systematically analyzing these factors, this review seeks to offer a better understanding of the key influences driving vaping intention among adolescents. These findings may support the development of prevention and intervention programs tailored to address the specific factors contributing to adolescents’ decisions to vape, ultimately reducing its prevalence and associated health risks [25].The practical implications of this review may also guide public health policies and educational campaigns targeting adolescents, parents, and educators.

## Materials and methods

This systematic review is prepared in accordance with the PRISMA (Preferred Reporting Items for Systematic Reviews and Meta Analyses) guideline. The objective of this review is to identify the predictors associated with vaping intention among adolescents. The PEO (population, exposure, outcome) mnemonic components were defined as follows:

- Population: Adolescents aged 10 to 19 years old.
- Exposure: Predictors associated with intention to use vapes among adolescents.
- Outcome: Vaping intention.

There are several definitions of intention used in prior research. Within the TPB, intention is a key construct that reflects an individual’s decision to exert effort to perform a behavior [26]. This framework emphasizes that intention is the most immediate predictor of actual behavior [24]. Previous smoking studies have also used susceptibility as a broader concept of intention [27, 28]. For instance, smoking intention has been defined as the lack of a firm commitment not to smoke among never-smokers, which has been shown to strongly predict future established smoking [15, 17, 29]. Similarly, within the context of vape use, susceptibility is defined as the absence of a firm decision not to use vapes [15, 30]. In this systematic review, intention is operationalized by incorporating susceptibility, as supported by prior studies. Therefore, intention was defined as an adolescent’s decision to use vapes, or their likelihood of doing so when offered by their best friends.

### Searching strategy

The literature search was conducted from April 1, 2024 to May 30, 2024, using the PubMed, CINAHL, Scopus and Science Direct databases. The search terms included: “Intention” OR “Susceptibility” OR “Willingness” OR “Decision” OR “Plan” AND “Vaping” OR “Electronic Cigarette” OR “E-cigarette” AND “Adolescent” OR “Youth” OR “Teen” AND “Predictor”. All retrieved articles were imported into an EndNote20 library.

### Eligibility criteria

The inclusion criteria were as follows: [1] publication in English; [2] original articles, including cohort, case-control, cross-sectional and clinical trials investigating the predictors of vaping intention; [3] study population consisting of adolescents; and [4] vaping intention or e-cigarette intention as an outcome. Exclusion criteria included qualitative studies and non-original articles, such as conference proceedings, perspective pieces, commentaries, opinion, reports, systematic review, and meta-analyses. Only articles published from 2017 to 2022 were selected to ensure relevance to the evolving landscape of adolescent vaping. This period marks as a significant shift in product design such as the introduction of USB-shaped devices with prefilled cartridges (pods), arrived on the market [11, 31]. These have influenced adolescent’s vape usage and intention. Additionally, key regulatory changes in late 2016, such as stricter US Food and Drug Administration (FDA) regulations and the European Union Tobacco Products Directive, reshaped vaping accessibility and marketing [32, 33]. By focusing on studies from 2017 onwards, we aimed to capture the most up-to-date evidence on vaping intention, ensuring that our findings are applicable to contemporary adolescent behavior and public health interventions.

### Study selection

Two independent reviewers screened the titles and abstracts of the retrieved studies based on the inclusion and exclusion criteria. Articles identified as potentially relevant were retained for full text review, which was conducted independently by three reviewers according to the same criteria. Any disagreements were resolved through group discussion among all the reviewers.

### Critical appraisal

Quality appraisal was conducted using the Mixed Method Appraisal Tool (MMAT). The MMAT evaluates the article quality, focusing on methodological criteria and core quality criteria relevant to its study design, especially when the review includes a mix of study designs. It covers following key areas of research quality including study design, appropriateness, data collection, data completeness, handling bias, analysis, integration and coherence.

### Data extraction and synthesis

Data were extracted and coded by two reviewers independently and then evaluated by a third and fourth reviewer. Eligible articles were analyzed in detail using the content analysis method without statistical testing. In this systematic review of 13 studies, several predictors of intended vape use were identified. We categorized the predictors in this review of vaping intention based on prior literature [31] and adapted theory of triadic influence (TTI), whereby the personal construct is further sub-categorized into sociodemographic, psychological and personal traits factors. All variables were finally classified into five pre-defined categories [34, 35]: [1] sociodemographic [2], psychological and personality [3], social [4], environmental, and [5] tobacco cognitive. Sociodemographic factors defined as individual’s intrapersonal and background characteristics that shape an adolescent’s experiences, opportunities, and behaviors, including their likelihood of engaging in vaping. Psychological and personality factors refer to an individual’s emotional, mental, and behavioral characteristics that influence their decision-making, risk-taking tendencies, and susceptibility to vaping. The social factors encompass the influence of an individual’s social environment, including connections and bonds with family, peers, and societal norms, that may shape their vaping-related behaviors. The environmental factors refer to external influences within the surrounding community, including media, marketing, and commercial availability, that shape perceptions and behaviors related to vape use. Tobacco cognitive influence refers to the thoughts, beliefs, and cognitive processes individuals have about tobacco or vape use, including their perceptions, attitudes, knowledge, and expectations regarding smoking or vaping. Factors that showed significance at the *p* ≤ 0.05 level were emphasized in a table.

## Results

**Fig. 1:**
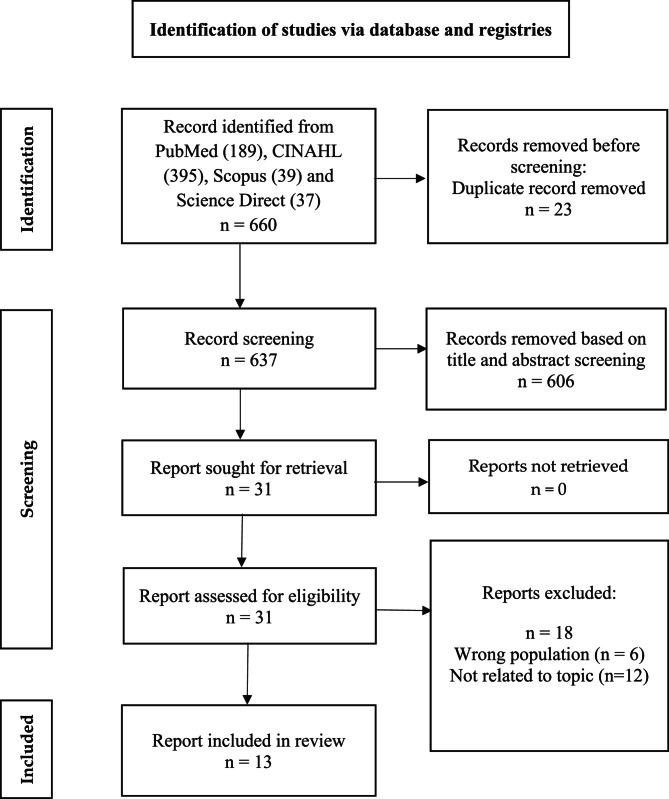
PRISMA flow diagram for the systematic review

The search yielded 189 articles from PubMed, 395 from CINAHL, 39 from SCOPUS and 37 from Science Direct, resulting in 660 unique hits. After rigorous selection screening, only 31 articles were included in the full-text assessment, as shown in the PRISMA flow diagram (Fig. 1). A descriptive summary of the studies included in this review, detailing study location and design, is presented in Table 1. The findings from 13 studies were included in this systematic review, as shown in Table 2. Of these, seven eligible articles were from US, one the from UK, two from Hong Kong, one from Spain, one from Poland and one from China. The analyzed articles were published between 2017 and 2022. Nine articles were cross-sectional studies, two articles were cohort studies, one article was a mixed methods study, and one was a controlled trial. Duplicate articles were initially removed using Endnote, followed by manual filtering of duplicate records. The sample size ranged from 468 to 40, 202 adolescents. The summary of the accepted articles is presented in Table 3..

**Table 1:** Summary of study location and study design

Authors	Study Location	Study Design
Pu & Zhang., (2017)	US	Cross-sectional
Kwon et al., (2018)	US	Cross-sectional
Leung et al., (2018)	Hong Kong	Cross-sectional
Patiño-Masó et al., (2019)	Spain	Cross-sectional
Kaleta et al., (2019)	Poland	Cross-sectional
Carey et al., (2019)	US	Cohort
Choi et al., (2020)	US	Cohort
Wang et al., (2020)	Hong Kong	Cross-sectional
Katz et al., (2020)	US	Control Trial
Simpson et al., (2020)	UK	Mixed Method
Tackett et al., (2021),	US	Cross-sectional
Kirkpatrick et al., (2022)	US	Cross-sectional
Dai et al., (2022)	China	Cross-sectional

**Table 2:** Predictors of vaping intention in adolescents

		Statistically significant association	
Predictors	Total analysis (*N*)	Significant analysis (*n*)	Direction of association
Sociodemographic factors			
Age	3	2	Positive
Gender	4	2	Positive, (Female > male)
Race/Ethnicity	3	3	Positive
Grade	2	2	Postive:1, Negative:1
Parent education	2	2	Positive
School performance	1	1	Positive
Substance use	4	4	Positive
Psychological and Personality factors			
Internalizing problem	1	1	Positive
Externalizing problem	1	1	Positive
Positive affect	1	1	Negative
Emotional problem	1	1	Negative
Sensation seeking	1	1	Positive
Rule breaker	1	1	Positive
Like frightening things	1	1	Positive
Prefer unpredictable friends	1	1	Positive
Social factor			
No curfew at home	1	1	Negative
Secondhand smoke/aerosol exposure	3	3	Positive
Friends vaping	3	2	Positive:1, Negative:1
Household vaping	3	2	Positive
Friend smoking	2	2	Positive
Household smoking	4	4	Positive:3, Negative:1
Family influence	3	1	Positive
Social norms	9	3	Positive:2, Negative:1
Environmental factors			
Exposure to vapes advertisement	7	6	Positive
Exposure to vapes marketing	6	6	Positive
Tobacco cognitive factors			
Perceived risk of vaping	8	6	Positive: 3, Negative: 3
Favorable perception of vaping	18	17	Positive
Attitudinal belief	9	8	Positive
Control belief	6	6	Positive

Table 2. summarizes predictors of vaping intention among adolescents. The first column lists the predictor variables, the second column (N) represents the number of studies that examined the predictor, the third column (n) indicates the number of studies that found a statistically significant association, and the final column specifies the direction of association. A ‘positive’ association means the predictor increased vaping intention, while a ‘negative’ association indicates a reduced likelihood of vaping intention.

### Vaping intention

This article presents 13 studies focused on vaping intention among adolescents. Most studies applied a consistent definition of vaping intention; however, differences arose in the measurement tools used. Some studies assessed vaping intention using a single-item measure, while others employed multi-item scales. Additionally, variations existed in the timeframe for assessing intention, with some studies examining short-term intention (e.g., within the next month or six months) and others focusing on long-term intention (e.g., within the next five years) [36, 37].

Potential predictors for intention to use vape in the future were categorized into five broad groups: socio-demographic, personality/psychological, social, environmental and tobacco cognitive factors. Most studies included in this review reported a significant effect on vaping intention. Table 2 summarizes the 39 predictors found to be statistically significantly associated with vaping intention in at least one study. In the following paragraphs the denominators representing the direction of association refer only to studies in which the direction of a statistically significant association was explicitly reported.

**Table 3.** Summary of accepted articles.

Author (Year): Country	Title	Study Design	Sample Size	Outcome/Method of Assessment	Predictors
Pu & Zhang., (2017): US	Exposure to advertising and perception, interest, and use of e-cigarettes among adolescents: findings from the US National Youth Tobacco Survey	Cross-sectional	12,595	Planned to use e-cigarette Self-administered questionnaire: 1 item to measure planned to use	a) **Environmental factors** Exposure to e-cigarette advertisement: • Via Internet (AOR = 1.61, 95% CI = 1.32–1.96, *p* < 0.05), • Via Newspaper/magazine (AOR = 1.30, 95% CI = 1.07–1.60, *p* < 0.05) • Via Stores (AOR = 1.32, 95% CI = 1.07–1.62, *p* < 0.05) • Via TV (AOR = 1.31, 95% CI = 1.07–1.59, *p* < 0.05)
Kwon et al., (2018): US	Predictors of youth e-cigarette use susceptibility in a US nationally representative sample	Cross-sectional	9853	Susceptibility to e-cigarette use Self-administered questionnaire: 2 items to measure susceptibility to use	a) **Sociodemographic factors** • Age: 15 to 17 years old (AOR = 1.16, 95% CI = 1.01–1.35, *p* < 0.05) • Race/Ethnicity: Hispanic white (AOR = 2.05, 95% CI = 1.78–2.36, *p* < 0.001) • Alcohol use: Ever user (AOR = 2.97, 95% CI = 1.49–5.92, *p* < 0.001) • Marijuana: Ever user (AOR = 1.59, 95% CI = 1.23–2.04, *p* < 0.001) • Other substance: Ever user (AOR = 1.38, 95% CI = 1.11–1.72, *p* < 0.01) b) **Tobacco cognitive factors** Attitudinal belief • Attitude towards advertisement: favorable (AOR = 2.22, 95% CI = 1.70–2.92, *p* < 0.001) Risk perception of e-cigarette • Addictiveness of e-cigarette: very addictive (AOR = 0.40, 95% CI = 0.29–0.54, *p* < 0.001), likely addictive (AOR = 0.56, 95% CI = 0.42–0.75, *p* < 0.001) • Perceived e-cigarette relative harmful: more harmful (AOR = 0.51, 95% CI = 0.36–0.71, *p* < 0.001), about the same (AOR = 0.50, 95% CI = 0.44–0.57, *p* < 0.001) c) **Psychological and Personality factors** • Internalizing problem: moderate (AOR = 1.19, 95% CI = 1.00-1.42, *p* < 0.05), high (AOR = 1.29, 95% CI = 1.06–1.56, *p* < 0.05) • Externalizing problem: moderate (AOR = 1.35, 95% CI = 1.17–1.56, *p* < 0.001), high (AOR = 1.71, 95% CI = 1.34–2.20, *p* < 0.001) • Rule breaker: strongly agree (AOR = 3.27, 95% CI = 2.17–4.91, *p* < 0.001), agree (AOR = 3.19, 95% CI = 2.44–4.17, *p* < 0.001), neither (AOR = 2.70, 95% CI = 2.11–3.45, *p* < 0.001), disagree (AOR = 1.96, 95% CI = 1.49–2.58, *p* < 0.001) • Like frightening things: disagree (AOR = 1.33, 95% CI = 1.09–1.63, *p* < 0.01) • Prefer unpredictable friends: strongly agree (AOR = 1.40, 95% CI = 1.01–1.96, *p* < 0.05), agree (AOR = 1.40, 95% CI = 1.08–1.81, *p* < 0.05) d) **Social factors** • Secondhand smoke exposure at home: cigarettes, cigars, cigarillos or filtered cigars (AOR = 1.27, 95% CI = 1.09–1.48, *p* < 0.05), smokeless or other tobacco only (AOR = 1.40, 95% CI = 1.07–1.84, *p* < 0.05) • No curfew presence at home (AOR = 0.84, 95% CI = 0.73–0.96, *p* < 0.05)
Leung et al., (2018): Hong Kong	Favorable perceptions of electronic cigarettes relative to cigarettes and the associations with susceptibility to electronic cigarette use in Hong Kong Chinese adolescents	Cross-sectional	40,202	Susceptibility to e-cigarette use Self-administered questionnaire: 2 items to measure susceptibility to use	a) **Tobacco cognitive factors** Favorable perception of e-cigarette • Perceived e-cigarette less likely to cause accidents such as fires and burns [Adjusted prevalence ratio (APR) = 1.85, 95% CI = 1.71-2.00, *p* < 0.001] • Perceived e-cigarette less harmful: to users (APR = 2.56, 95% CI = 2.33–2.80, *p* < 0.001) • Perceived e-cigarette less harmful: to others (APR = 2.29, 95% CI = 2.04–2.57, *p* < 0.001) • Perceived e-cigarette more attractive (APR = 3.58, 95% CI = 3.13–4.10, *p* < 0.001) • Perceived e-cigarette more chic (APR = 2.84, 95% CI = 2.53–3.18, *p* < 0.001) • Perceived e-cigarette easier for minors to buy (APR = 2.73, 95% CI = 2.47–3.02, *p* < 0.001) • Perceived e-cigarette easier to use: at home unnoticed (APR = 2.43, 95% CI = 2.14–2.75, *p* < 0.001) • Perceived e-cigarette easier to use: at school unnoticed (APR = 2.28, 95% CI = 2.01–2.59, *p* < 0.001) • Perceived e-cigarette more environmentally friendly (APR = 2.15, 95% CI = 1.91–2.42, *p* < 0.001) • Perceived e-cigarette more convenient (APR = 2.16, 95% CI = 1.95–2.40, *p* < 0.001) • Perceived e-cigarette cleaner (APR = 2.25, 95% CI = 2.02–2.50, *p* < 0.001) • Perceived e-cigarette use in children is better accepted: by parents (APR = 3.50, 95% CI = 3.01–4.08, *p* < 0.001) • Perceived e-cigarette use in children is better accepted: by schools (APR = 3.29, 95% CI = 2.72–3.98, *p* < 0.001)
Patiño-Masó et al., (2019): Spain	Predictors of intentions to use cigarettes and electronic-cigarettes among high school students	Cross-sectional	468	Intention to use e-cigarette in the future Self-administered questionnaire: 8 items to measure intention use	a) **Sociodemographic factors** • Self-report smoking tobacco: some time in their life (OR = 0.33, 95% CI = 0.14–0.74) • Current e-cigarette user (OR = 0.15, 95% CI = 0.04–0.61) b) **Social factors** • Friends use e-cigarette (OR = 0.22, 95% CI = 0.07–0.65, *p* < 0.01)
Kaleta et al., (2019): Poland	Predictors of E-Cigarette Use Susceptibility: A Study of Young People from a Socio-Economically Disadvantaged Rural Area in Poland	Cross-sectional	1,693	Susceptibility to e-cigarette use Self-administered questionnaire: 1 item to measure intention not to vape	a) **Sociodemographic factors** • School grade: secondary school (AOR = 1.12, 95% CI = 0.55-4.00) • Alcohol use: moderate (AOR = 2.94, 95% CI = 1.50–5.76, *p* < 0.001), binge (AOR = 2.19, 95% CI = 1.25–3.82, *p* < 0.01) • Current smoker (AOR = 14.05, 95% CI = 3.30-59.81, *p* < 0.001) • Mother’s education: Medium (AOR = 2.72, 95% CI = 1.48–5.02, *p* < 0.01), High (AOR = 2.71, 95% CI = 1.15–6.39, *p* < 0.05) • Father’s education: Medium (AOR = 0.67, 95% CI = 0.34–1.07), High (AOR = 1.97, 95% CI = 0.76–5.09) b) **Social factors** • Parental smoking (AOR = 3.03, 95% CI = 1.61–5.69, *p* < 0.001) • Friends smoking (AOR = 1.98, 95% CI = 1.06–3.70, *p* < 0.05) c) **Tobacco cognitive factors** Favorable perception of e-cigarette • Perceived e-cigarette less harmful (AOR = 1.80, 95% CI = 1.04–3.10, *p* < 0.001) • Perceived girls who use e-cigarette more attractive (AOR = 4.08, 95% CI = 2.05–8.10, *p* < 0.001)
Carey et al., (2019): US	Understanding susceptibility to e-cigarettes: A comprehensive model of risk factors that influence the transition from non-susceptible to susceptible among e-cigarette naïve adolescents	Cohorts	1,369	Susceptibility to e-cigarette Self-administered questionnaire: 3 items to measure susceptibility to e-cigarette	a) **Sociodemographic factors** • Other substance use (OR = 7.81, 95% CI = 2.66–22.95) for 11–12 years old, other substance use (OR = 3.92, 95% CI = 1.19–12.93) for 13–14 years old • School performance: low academic (OR = 12.98, 95% CI = 1.58-106.84) for 13–14 years old b) **Tobacco cognitive factors** Risk perception of e-cigarette • E-cigarette are harmful (OR = 0.69, 95%CI = 0.49–0.95) for 13–14 years old c) **Social factors** • Family influence (OR = 4.72, 95% CI = 1.09–20.56) for 11–12 years old • Social norms - okay to use (OR = 6.69, 95% CI = 1.24–36.01) for 13–14 years old • Social norms - common to use e-cigarette (OR = 1.42, 95% CI = 1.14–1.78) for 13–14 years old • Social norms - would date e-cigarette user (OR = 0.52, 95% CI = 0.31–0.88) for 13–14 years old d) **Psychological and Personality factors** • Positive affect (OR = 0.61, 95% CI = 0.46–0.80) for 13–14 years old • Emotional problem (OR = 1.11, 95% CI = 0.97–1.27) for 13–14 years old • Sensation seeking (OR = 1.45, 95% CI = 1.06–1.99) for 13–14 years old
Choi et al., (2020): US	Exposure to Multimedia Tobacco Marketing and Product Use Among Youth: A Longitudinal Analysis	Cohort	10,081	Susceptibility for tobacco product: cigarettes, e-cigarettes, traditional cigars, cigarillos, filtered cigars, pipe, hookah, smokeless tobacco, and snus Self-administered questionnaire: 4 items to measure susceptibility to use	a) **Sociodemographic factors** • Gender: Female (AOR = 1.30, 95% CI = 0.95–1.78) • Race/Ethnicity: Hispanic or Latino (AOR = 1.21, 95% CI = 0.80–1.81), non-Hispanic black (AOR = 1.29, 95% CI = 0.79–2.10), non-Hispanic other (AOR = 0.74, 95% CI = 0.36–1.51) • Grade: High school or more (AOR = 0.96, 95% CI = 0.70–1.31) • Parent education: High school (AOR = 1.40, 95% CI = 0.84–2.35), College (AOR = 0.67, 95% CI = 0.27–1.64), Bachelor’s degree (AOR = 0.73, 95% CI = 0.37–1.43), Advanced degree (AOR = 1.45, 95% CI = 0.78–2.69) b) **Social factors** • Living with tobacco user (AOR = 1.25, 95% CI = 0.78–2.69) c) **Environmental factors** • Received tobacco coupon (AOR = 3.83, 95% CI = 2.56–5.74) • Online engagement score: Score 1 (AOR = 1.62, 95% CI = 1.04–2.54), Score 2+ (AOR = 3.45, 95% CI = 1.74–6.83)
Wang et al., (2020): Hong Kong	Exposure to e-cigarette advertising, attitudes, and use susceptibility in adolescents who had never used e-cigarettes or cigarettes	Cross-sectional	8704	Susceptibility to e-cigarette use Self-administered questionnaire: 2 items to measure susceptibility to use	a) **Environmental factors** • Exposure to e-cigarette advertisement (AOR = 1.78, 95% CI = 1.40–2.20, *p* < 0.001) b) **Tobacco cognitive factors** Attitude towards e-cigarette • Uncertain that e-cigarette use harm health (AOR = 1.57, 95% CI = 1.28–1.93, *p* < 0.001) • Tolerant attitudes towards e-cigarette use (AOR = 3.30, 95% CI = 2.68–4.06, *p* < 0.001) • Tobacco industry is respectable (AOR = 1.70, 95% CI = 1.39–2.07, *p* < 0.001)
Katz et al., (2020): US	High School Youth and E-cigarettes: The Influence of Modified Risk Statements and Flavors on E-cigarette Packaging	Randomized control trial	715	Behavior Intention to vape Self-administered questionnaire: 1 item to measure intention to vape	a) **Tobacco cognitive factors** Risk perception of e-cigarette • Perceived risk of e-cigarette: F (1, 605) = 205.84, *p* < 0.001
Simpson et al., (2020): UK	Employing the theory of planned behaviour to design an e-cigarette education resource for use in secondary schools	Mixed-method	1511	Intention to use e-cigarette Self-administered questionnaire: 3 items to measure intention to use	a) **Social factors** Normative belief (F^(6, 1456)^ = 140.8, *p* < 0.001) • friends encouraging the use of e-cigarette (β = 0.090, *p* < 0.001: spc^2^ = 0.099) • family encouraging the use of e-cigarette (β = 0.040, *p* = 0.04: spc^2^ = 0.005) • parents encouraging the use of e-cigarette (β = 0.037, *p* = 0.005: spc^2^ = 0.005) • medical professionals encouraging the use of e-cigarette (β = 0.037, *p* < 0.001: spc^2^ = 0.011) b) **Tobacco cognitive factors** Attitude belief (F^(8, 1438)^ = 106.4, *p* < 0.001) • More fun to use (β = 0.138, *p* < 0.001: spc^2^ = 0.18) • Cheaper to buy (β = 0.054, *p* < 0.001: spc^2^ = 0.033) • Safer to use than tobacco cigarettes (β = 0.029, *p* < 0.001: spc^2^ = 0.009) • Less likely to get young people into trouble with parents (β = 0.031, *p* < 0.001: spc^2^ = 0.030) Control belief (F^(11, 1422^) = 148.6, *p* < 0.001) • parental permission to use e-cigarettes (β = 0.291, *p* < 0.001: spc^2^ = 0.112) • getting into trouble with parents for using (β = 0.176, *p* < 0.001: spc^2^ = 0.046) • curiosity to try (β = 0.112, *p* < 0.001: spc^2^ = 0.018) • owning one (β = 0.083, *p* < 0.001: spc^2^ = 0.012) • friends who are using e-cigarettes (β = 0.047, *p* = 0.054: spc^2^ = 0.002) • adhering to the law (β = 0.041, *p* = 0.021: spc^2^ = 0.003)
Tackett et al., (2021): US	Adolescent Susceptibility to E-Cigarettes: An Update From the 2018 National Youth Tobacco Survey	Cross-sectional	12,439	Susceptibility to use e-cigarettes Self-administered questionnaire: 4 items to measure susceptibility to e-cigarette	a) **Sociodemographic factors** • Age: 14–15 years old (AOR = 1.04, 95% CI = 0.93–1.17), 16–17 years old (AOR = 0.88, 95% CI = 0.75–1.03) • Gender: Female (AOR = 1.19, 95% CI = 1.04–1.36) • Race/Ethnicity: Hispanic (AOR = 1.30, 95% CI = 1.13–1.49), NH-Black (AOR = 0.72, 95% CI = 0.61–0.86), others (AOR = 1.04, 95% CI = 0.86–1.27) b) **Tobacco cognitive factors** Perceived risk of e-cigarette • Perceived harm of e-cigarette: No harm (AOR = 3.50, 95%CI = 2.68–4.58), Less harm (AOR = 4.90, 95%CI = 4.09–5.89), Some harm (AOR = 2.20, 95%CI = 1.92–2.52) • Addictiveness of e-cigarette: Less addictive (AOR = 1.43, 95%CI = 1.21–1.68), More addictive (AOR = 1.15, 95%CI = 0.95–1.38) Favorable perception of e-cigarette • ease of purchasing: Easy (AOR = 1.40, 95% CI = 1.23–1.59) c) **Social factors** Household use of tobacco product • Combustible tobacco at home (AOR = 1.12, 95% CI = 0.96–1.30) • Non-combustible tobacco other than e-cigarette in the home (AOR = 0.90, 95% CI = 0.70–1.17) • E-cigarette at home (AOR = 1.48, 95% CI = 1.15–1.90)
Kirkpatrick et al., (2022): US	Recognition of cartoon-based e-cigarette-related marketing is associated with e-cigarette use among adolescents	Cross-sectional	1,743	Susceptibility to use e-cigarettes in the future Self-administered questionnaire: 2 items to measure susceptibility to e-cigarette	a) **Environmental factors** • Cartoon-based marketing e-cigarette (OR = 2.25, 95% CI = 1.48–3.43, *p* < 0.001)
Dai et al., (2022): China	Social environment exposure to electronic cigarettes and its association with e-cigarette use among adolescents in Shanghai, China	Cross-sectional	16,123	Intention to use e-cigarette Self-administered questionnaire: 2 items to measure intention to use	a) **Social factors** • Parent’s e-cigarette use (AOR = 1.99, 95% CI = 1.53–2.59, *p* < 0.001) • Friend’s e-cigarette use (AOR = 3.55, 95% CI = 2.92–4.32, *p* < 0.001) • Second-hand e-cigarette aerosol (AOR = 2.68, 95% CI = 2.23–3.21, *p* < 0.001) b) **Environmental factors** • E-cigarette sales exposure: one source (AOR = 1.62, 95% CI = 0.28-2.04, *p* < 0.001), two or more sources (AOR = 2.73 95% CI = 2.15–3.46, *p* < 0.001) • E-cigarette information exposure: two or more sources (AOR = 1.98, 95% CI = 1.53–2.55, *p* < 0.001

### Predictors of vaping intention

#### Sociodemographic factors

Sociodemographic factors were examined in six studies. However, the number of significant variables included in each study differ as shown in Table 2. Positive relationships with intention were found for age (2/3 studies), gender (2/4 studies), ethnicity (3/3), grade (2/2), parent education (2/2), school performance (1/1) and substance use (4/4). A Population Assessment of Tobacco and Health (PATH) study among adolescents aged 12–17 years reported that older age (15–17 years old) significantly predicted intention (AOR = 1.16, 95% CI = 1.01–1.35, *p* < 0.05) [30]. Conversely, a study using a 2018 NYTS for the same age group found that older adolescents (16–17 years old) were a protective factor (AOR = 0.88, 95%CI = 0.75–1.03), while younger adolescents (14–15 years old) were at higher risk for vaping intention (AOR = 1.04, 95%CI = 0.93–1.17) [38].

Differences in school grade showed only a small effect on predicting intention [39, 40]. Regarding gender, both studies identified female as a stronger predictor for vaping intention compared to males [38, 39]. In context of ethnicity, Hispanic individuals were identified as being at higher risk in both studies compared to non-Hispanic White individuals. However, findings for non-Hispanic black individuals were mixed, with reported association of [(AOR = 1.29, 95% CI = 0.79–2.10) and (AOR = 0.72, 95%CI = 0.61–0.86)], respectively across studies. Similarly, mixed findings were reported for others ethnic groups [(AOR = 0.74, 95% CI = 0.36–1.51) and (AOR = 1.04, 95%CI = 0.86–1.27)] [38, 39]. Parental education also played a role in predicting vaping intention. A study conducted by Kaleta et al., (2019) found that adolescents with parents who had higher education levels were 2.71 times and 1.97 times more likely to exhibit vaping intention compare to those with parents with lower education levels (Mother’s education: AOR = 2.71, 95%CI = 1.15–6.39) and (Father’s education: AOR = 1.97, 95%CI = 0.76–5.09) [40]. Similarly, from the US indicated that adolescents with parents who had higher education levels had greater odds of intending to vape compared to those with parents with lower educational levels (AOR = 1.45, 95% CI = 0.78–2.69) [39]. Additionally, one study identified a significant association between low academic performance and vaping intention in adolescents (OR = 12.98, 95%CI = 1.58-106.84) [28]. Substance use also emerged as a significant predictor of vaping intention. Current cigarette smoking (AOR = 14.05, 95%CI = 3.30-59.81), alcohol use (AOR = 2.94, 95%CI = 1.50–5.76) and marijuana use were strongly associated with increase vaping intention [40]. Kwon, Seo [30] similarly identified this substance use factors as predictor of vaping intention.

#### Psychological and personality factors

Personality/psychological factors were investigated in two studies, all of which reported statistically significant associations with vaping intention. The examined psychological variables included internalizing problems, externalizing problems, positive affect, emotional problem, sensation seeking, rule-breaker, like frightening things and prefer unpredictable friends. Internalizing problems refer to inwardly directed emotional or psychological distress such as anxiety, depression, and traumatic distress. Externalizing problems are characterized by outwardly directed behaviors, including aggression, impulsivity, and conduct disorder [41, 42]. A negative association was found only between intention and positive affect (1/1 studies). According Kwon, Seo [30], moderate to high internalizing problems significantly increased vaping intention compared to low levels. This finding is supported by Carey, Rogers [28], who reported that positive affect reduced the risk of vaping intention (OR = 0.61, 95%CI = 0.46–0.80). Additionally, moderate to high levels of externalizing problems were also found to predict vaping intention [30].

#### Social factors

Social factors were examined in seven studies. However, the number of significant variables included in each study differ as shown in Table 2. Most studies investigated adolescent’s social smoking or vaping environment factors either related to friends use or household (parents/siblings) use. Family influence, parental smoking and friend’s vape use are strongly associated with higher odds of vaping intentions. Conversely, social norms that participant would date someone who use vapes (OR = 0.52, 95% CI = 0.31–0.88) and no curfew presence at home (AOR = 0.84, 95% CI = 0.73–0.96, *p* < 0.05) were protective factor towards vaping intention [28, 30].

#### Environmental factors

Environmental influences on vaping were examined in five studies, all of which studies reported a statistically significant relationship with vaping intention. In all five studies, exposure to vaping advertisement and marketing, was positively associated with intention. Study in China found exposure to all types of vaping advertisement media was associated with vaping intention (AOR = 1.78, 95% CI = 1.40–2.20, *p* < 0.001) [43]. A study among youth in US by Pu & Zhang., (2017) reported higher odds of online exposure via internet compared to other offline sources (AOR = 1.61, 95% CI = 1.32–1.96, *p* < 0.05). Similar findings were reported by Choi, Rose [39], where adolescents with higher online engagement scores for tobacco product advertisement in more than two forms had increased odd of vaping intention (AOR = 3.45, 95% CI = 1.74–6.83). Exposure to cartoon based-vapes marketing also yielded a significant associated to vaping intention among adolescents [44]. Study among US adolescent by Choi, Rose [39] found vapes marketing through discount coupons showed higher odds of vaping intention (AOR = 3.83, 95% CI = 2.56–5.74).

#### Tobacco cognitive factors

Tobacco cognitive factors were examined across eight studies, all of which yielded a statistically significant relationship with vapes intention in at least one study. Perceived less risks towards vapes, favorable perceptions, favorable attitude, normative and control beliefs of vaping were positively associated with vaping intention. Adolescents who perceived vapes are more attractive (APR = 3.58, 95% CI = 3.13–4.10, *p* < 0.001), vapes use in children better accepted by parents (APR = 3.50, 95% CI = 3.01–4.08, *p* < 0.001) and by schools (APR = 3.29, 95% CI = 2.72–3.98, *p* < 0.001) reported higher prevalent towards vapes intention [45]. Likewise, vaping intention was significantly and strongly predicted with perceive girls used vapes as attractive (AOR = 4.08, 95% CI = 2.05–8.10) and perceived vapes as less harm (AOR = 4.90, 95% CI = 4.09–5.89) [38, 40]. Whereas study by Kwon, Seo [30] among US adolescents found perceive addictiveness and relative harmful of vaping as protective factors towards intention to vapes.

### Risk of bias

The authors conducted quality appraisal of all ten studies using the Mixed Method Appraisal Tool (MMAT), version 2018 [46]. The MMAT allows for simultaneous evaluation of all empirical literature (i.e., qualitative, quantitative, and mixed methods studies [47]. This appraisal tool has been shown to be efficient, user friendly, and has high intraclass correlation [48]. Two reviewers were independently assessed the quality of each study. The quality of each selected article was evaluated based on methodological criteria, which included five core quality criteria. Eligible articles were analysed in detail using the content analysis method without any statistical tests. Where scores differed, discrepancies were resolved through discussion. The details of this assessment for the studies selected are reported in Table 4. All selected studies met the good methodological standard outlined by MMAT, and therefore are included in this study.

**Table 4:** The details of the MMAT assessment

Author	Type of Study	1.1	1.2	1.3	1.4	1.5
		Are the participants representative of the target population?	Are measurements appropriate regarding both the outcome and intervention (or exposure)?	Are there complete outcome data?	Are the confounders accounted for in the design and analysis?	During the study period, is the intervention administered (or exposure occurred) as intended?
Pu & Zhang., (2017)	Quantitative non-randomized	Yes	Yes	Yes	Yes	No
Kwon et al., (2018)	Quantitative non-randomized	Yes	Yes	Yes	Yes	No
Leung et al., (2018)	Quantitative non-randomized	Yes	Yes	Yes	Yes	No
Patiño-Masó et al., (2019)	Quantitative non-randomized	Yes	Yes	Yes	Yes	No
Kaleta et al., (2019)	Quantitative non-randomized	Yes	Yes	Yes	Yes	No
Carey et al., (2019)	Quantitative non-randomized	Yes	Yes	Yes	Yes	No
Choi et al., (2020)	Quantitative non-randomized	Yes	Yes	Yes	Yes	No
Wang et al., (2020): Hong Kong	Quantitative non-randomized	Yes	Yes	Yes	Yes	No
Tackett et al., (2021)	Quantitative non-randomized	Yes	Yes	Yes	Yes	No
Kirkpatrick et al., (2022)	Quantitative non-randomized	Yes	Yes	Yes	Yes	No
Dai et al., (2022)	Quantitative non-randomized	Yes	Yes	Yes	Yes	No
**Author**	**Type of study**	**2.1**	**2.2**	**2.3**	**2.4**	**2.5**
		Is randomization appropriately performed?	Are the groups comparable at baseline?	Are there complete outcome data?	Are outcome assessors blinded to the intervention provided?	Did the participants adhere to the assigned intervention?
Katz et al., (2020)	Quantitative randomized controlled trials	Yes	Yes	Yes	Yes	Yes
**Author**	**Type of study**	**3.1**	**3.2**	**3.3**	**3.4**	**3.5**
		Is there an adequate rationale for using a mixed methods design to address the research question?	Are the different components of the study effectively integrated to answer the research question?	Are the outputs of the integration of qualitative and quantitative components adequately interpreted?	Are divergences and inconsistencies between quantitative and qualitative results adequately addressed?	Do the different components of the study adhere to the quality criteria of each tradition of the methods involved?
Simpson et al., (2020)	Mixed method	Yes	Yes	Yes	Yes	Yes

## Discussion

This review summarized the existing evidence on factors influencing vaping intention among adolescents, focusing specifically on the predictors. Understanding these risk factors is a crucial phase in developing effective strategies to address the anticipated future challenge of improving public health by preventing vaping intention, as intention predicts behavior. This is an essential activity in combating the global tobacco epidemic, as outlined in the World Health Organization (WHO) Framework Convention on Tobacco Control (FCTC) in the Article 12, which encourage public education, awareness and prevention strategies to deter initiation. These efforts contribute to a comprehensive approach to tackling the challenge of vape use. In this systematic review of 13 studies, several predictors of intended vape use were identified and further classified into five broad pre-determined categories: [1] sociodemographic [2], personality/psychological [3], social [4], environmental and [5] tobacco cognitive factors. During the review, specific significant predictors were identified and classified according to the above categories.

### Predictors of vaping intention

#### Sociodemographic factors

Sociodemographic factors such as gender, age, race/ethnicity, academic performance, parental education and substance use have been found to influence vaping intention.

a. Gender.

Several studies consistently have reported that female adolescents have higher odds of intending to vape. This trends is supported by National Youth Tobacco Survey data by Margolis, Thakur [49] and findings from Greece, where male students aged 13 to 15 years were less likely to express vaping intentions compared to female students [50]. In Malaysia, the National Health and Morbidity Survey (NHMS) 2022 revealed that among female teenagers, the prevalence of vaping is nearly four times higher than the cigarette smoking. This contrast suggests a growing susceptibility to vaping within this subgroup, especially when compared to the declining trend in cigarette use [4]. This finding may help explain why female adolescents, despite historically lower rates of cigarette smoking than males, now demonstrate higher odds of intending to vape. However, Carey, Rogers [28] found no association between gender and vaping intention. The variation in these findings may be due to smaller sample size, which could have limited the ability to detect significant gender differences, even if an association existed. Larger studies, such as those utilizing national survey datasets [38, 39], may capture more subtle trends in gender differences, while smaller-scale studies might suffer from higher variability. Additionally, studies that employed more sensitive measures such as multi-item validated scales capturing various dimensions of vaping intention were more likely to detect subtle associations compared to those using single-item question.

Earlier data prior to 2019, identified males as more susceptible to vaping than females [51, 52]. This shift has been observed in two national US surveys and became more noticeable during the COVID-19 pandemic, when female adolescents reported higher levels of sadness and hopelessness than males, potentially contributing to increased intention to vapes initiation and use among them [53]. Longitudinal study also suggest that depressive symptoms may increase the likelihood of future vape use [54]. Other explanation for this shift is that female may perceive vaping as less harmful than other tobacco products, making them more motivated intending to initiate vaping [55]. Research indicates that females are more likely to vape due to stress relief, trying to lose weight, and product appeal, including multiple flavors, concealability, and vape tricks [56, 57]. Whereas males tend to be driven by nicotine dependence and tank style devices [55]. Al-Hamdani, Hopkins [58] found that vapes trick were significantly more important for females compared to males, who placed greater value on experiencing nicotine rush from vaping. Other possible explanation is that the vaping industry has employed marketing strategies via social networks that resonate with young females in the forms of advertisements and paid influencer by reviving the old techniques around vaping [59].

For example, JUUL’s early campaigns in 2015 featured vibrant imagery of smiling, stylish young women, promoting the product as trendy and socially appealing. Other brands like Krave and RELX used advertising strategies reminiscent of fashion and beauty marketing, associating vaping with personal style and sophistication. Furthermore, e-cigarette companies such as NJOY have sponsored high-profile events like New York Fashion Week to further align their products with glamour and femininity [60]. These marketing tactics may reinforce perceptions that vaping is stylish, empowering, and socially desirable, contributing to increased susceptibility and intention among adolescent females. Therefore, many females consider vaping to be trendy and fear missing out if they do not engage, especially if their friends are vaping [61].

b. Age.

The association between age and vaping intention has shown mixed finding in existing literature. A study in California found that adolescents and young adults aged 15 to 20 years old were more likely to have vaping intention compare to adults aged 21–29 years [62]. Conversely, a study among naïve adolescents in Malaysia found that age was not significantly associated with vaping intention [63]. This discrepancy may be due to differences in tobacco control regulations. In Malaysia, purchasing and using any tobacco substitute product for individuals under the age of 18 is not illegal until recently, when a new law (Control of Smoking Products for Public Health Act 2024, Act 852) was passed in 2024 [64]. In contrast, most Western countries have long-standing legislation restricting the use of tobacco and nicotine product among adolescents [65].

c. Ethnicity.

Past research has shown that race and ethnicity are significant factors associated with vaping intention [49]. A study conducted in US reported a higher likelihood of vaping intention among Hispanic-White individuals. A similar finding, observed by Ebrahimi Kalan, McKelvey [66], highlighted that Hispanic-White were more likely to intend to vape than non-Hispanic White individuals. In contrast, nationally representative data on vaping among youth indicate that non-Hispanic White adolescents have the highest rates of vape use [67]. The current review demonstrates that different patterns can emerge when considering regional data. Regional studies show that vapes use among Hispanic youth differs by location. For example, data from Utah found that Hispanic/Latino youth were more likely to have vaped in their lifetime and in the past 30 days compared to non-Hispanic White youth. This suggests that local culture and socioeconomic factors influence vaping intentions across racial and ethnic groups [68].

d. Academic performance.

The strong association between low academic performance and vaping intention in this review [28] aligns with earlier study [69], students with poor academic performance had higher intention to vape. This is further supported by study analyzing data from 2015 to 2019 National Youth Risk Behavior identified positive association between substance use, including vape use and poor academic performance [70]. This relationship may suggest students struggling in schools are more likely to engage in risky behavior, including vaping.

e. Cigarette smoking.

Our review also showed cigarette smoking emerged as a significant predictor of vaping intention [40]. This is supported by a longitudinal study by Morello, Perez [71] found that the predictors of tried vaping is being a current smoker. One in five students who were current smokers and had not tried vaping at baseline tried vaping at follow-up. The association suggests that adolescent who already smoke may perceive vaping as an alternative nicotine source, either for harm reduction strategy or to quit smoking [71]. This transition to vaping could be an initial step towards smoking cessation, but failing to quit might expose them to be a dual use, which poses greater health risks. Study has shown that the odd of diseases associated with dual use are higher than those associated with exclusive cigarette smoking for all outcomes, including cardiovascular disease, stroke, metabolic dysfunction, asthma, chronic pulmonary disease and oral disease [72].

#### Psychological and personality factors

In this review, vaping intention were significantly associated with both moderate to high internalizing and externalizing problems [30]. This aligns with previous study from the Population Assessment Tobacco Health (PATH) study conducted in US [42]. According to PATH study, higher levels of both internalizing and externalizing problems were associated with an increased risk of initiating exclusive vaping. While the reasons for this association were not explicitly tested in the reviewed studies, prior research indicate adolescents experienced a wide variety of stressor during their high school years, therefor may be drawn to behaviors that they perceive as offering stress relief benefits [73]. A study among US youths also found that psychological distress, such as anxiety, depression, loneliness and stress, was a significant predictor of the intention to use vapes [74]. Future studies should explore whether stress-related vaping intentions are driven by perceived coping benefits or other external influences.

Personality traits such as sensation seeking have been found to significantly predict vaping intention. Consistent with previous research [75], our review found that, higher sensation seeking scores among 8th grade were associated transition to intent to vaping within 12 months [28]. This also aligns with past studies showing that sensation seekers are drawn to behaviors perceived as exciting and increase the risk intends towards negative behavior such tobacco and vape use [75, 76]. Sensation seeking is a personality trait characterized by a desire for new, varied, and intense experiences, coupled with a willingness to take risks to obtain them. A study conducted among Indonesian adolescents and young adults found that individuals with high sensation-seeking tendencies are more likely to experiment with regularly use vapes, driven by the desire for new and thrilling experiences​ [77]. This suggests that preventive interventions should target personality traits such as sensation seeking to reduce susceptibility to vaping among adolescents.

#### Social factors

Social factors, including family influence, parental smoking, peer vaping/smoking, exposure to secondhand smoke/aerosol and social norms, play a crucial role in shaping adolescents’ intentions to vape. This review found that parental and peer vaping behavior were significant predictors of vaping intention, with most studies highlighting a positive association between exposure to vaping in social settings and increased intentions to vape use. Research in Australia supports these findings, showing that adolescents exposed to vaping by parents or family members were more likely to initiate vaping themselves [78]. Similarly, a meta-analysis of 58 studies found that the likelihood of smoking initiation doubled if a parent figure smoked [79], suggesting that parental modeling of tobacco-related behaviors extends to vaping as well. Having parents who smokes significantly increases the risk of smoking or vaping intention in childhood and adolescence. Therefore, parents are essential figures in fostering anti-vaping behavior among adolescents at home. Trucco, Cristello [80] found that positive parental guidance and negative attitude towards vaping were negatively associated with adolescents vaping intentions. In turn, lower vaping intentions led to reduced actual vape use. These findings highlight the importance of parental influence in preventing adolescent vaping through guidance and a clear anti-vaping attitude.

Peer influence emerged as another strong predictor of vaping intention, with multiple studies confirming that adolescents with friends who vape had higher odds of intending to vape [81–83]. Our finding that among adolescents aged 13 to 18 years old, who have intention to use vapes, were 3.55 times more likely to have best friends who are vaping, suggesting that peer influence potentially play a role in adolescent vape use. This result is consistent with a cohort study in US that emphasizes the role of peer influence in adolescents vape intention and initiating at follow-up [84]. Our review also found social norms surrounding adolescent like it’s okay to use vapes, it’s common to use vape, would date vape user and encourage use by close people (parents, family, friends) positively associated with intention to vapes. Adolescents in particular look to their peers to shape normative beliefs, and interpret information related to risky behavior [85]. According to multiple health behavior theories and models [24, 86], perceived peer approval and exposure to substance use are critical factors influencing the early stages of substance use during adolescence. The findings of this review align with these theoretical perspectives, demonstrating that peer vaping behaviors and social acceptance of vaping significantly shape adolescents’ intention to vape. This suggests that modifying social norms surrounding vape use and increasing awareness of its risks should be key components of public health efforts to counter the rising trend of youth vaping.

#### Environmental factors

Environmental factors, particularly exposure to vape advertisements, and marketing strategies, significantly influence adolescents’ intentions to use vapes. This review found that all studies examining environmental influences reported a statistically significant association between exposure to vaping advertisements and increased vaping intention. Our findings demonstrated that exposure to all types of vaping advertisement media, whether on broadcast, online, print, outdoor, or point-of-sale in stores, were significantly linked to a higher likelihood of vaping intention [43]. A US based study found that adolescents who frequently encountered online vaping advertisements had significantly higher odds of intending to vape compared to those exposed through traditional media sources [87]. The impact of social media and digital engagement further amplifies this association. Choi, Rose [39] reported that adolescents with high engagement in online tobacco advertisements across multiple platforms were over three times more likely to express vaping intentions. This may be explained by adolescent’s extensive use of social media platforms, therefore increase frequency of exposure to its content featuring vaping advertisement and products. A study in California among high school students further supported this relationship, in which frequent use and frequent exposure to tobacco content on TikTok is strongly associated with adolescent increased risk of intend initiation of vaping [88]. Additionally, exposure to cartoon-based vape marketing, which often targets younger audiences, was found to be a strong predictor of vaping intention [44]. This suggests that the youth-friendly appeal of vaping advertisements, often depicting flavored products and social benefits, contributes to increased susceptibility. Beyond direct advertisements, marketing tactics such as discount coupons and financial incentives also influenced vaping intention. A study among U.S. adolescents found that receiving discounts or promotional offers significantly increased the likelihood of vaping intention [39]. These attractive marketing tactics have provided the opportunity to vaping to become more accessible and affordable to the youngster, making them more desirable to try and initiate it ​ [19]. The findings from this review emphasize the need for stricter regulations on vapes marketing, particularly on digital and social media platforms, where adolescents are highly susceptible to exposure.

#### Tobacco cognitive factors

The relationship between tobacco cognitive influence and vaping intention among adolescents have also been observed in a study conducted in US [89]. Tobacco cognitive influence refers to cognitive processes and beliefs individuals hold about tobacco or vape use, including perceptions, attitudes, knowledge and expectations regarding smoking/vaping and its consequences [31]. The findings of this review indicate that adolescents risk perception, favourable perception towards vaping, attitudinal belief and control belief are significant factors influencing vaping intention. Adolescents who perceived vaping as addictive and relatively harmful were less likely to intend to vape, suggesting the heightened awareness of the risks serves as a protective factor [30]. Conversely, those who perceived vaping as less addictive and held more favourable views such as believing vapes are attractive, easily accessible for minors to buy, convenient to use unnoticed and more socially acceptable, were more likely to develop an intention to vape [45, 82]. These results are consistent with study on risk perception and risk taking behavior, which suggest that individual who perceived lower risk perception influences risk-taking behavior, where individuals who perceive lower risks are more likely to engage in risky behaviors, including vaping [90]. Trumbo and Kim [91] also found that positive attitudes towards vaping were associated with a greater likelihood of intending vaping in the near future. Similarly, a study conducted in UK reported that attitudes influenced both the intention among ever vape and never vape [92]. Positive attitude towards vaping are often shaped by product related features, such as being more fun to use, cheaper to buy, perceived as safer, and less likely to cause trouble with parents [37]. These features contributes to adolescents motivation and intend to try vapes [62]. Control beliefs, which refers to an individual’s perception of their ability to control their behavior, were also found to play a role in vaping intention. Social and environmental facilitators, such as parental permission and peer endorsement, significantly shape these beliefs [92]. Adolescents who receive explicit or implicit approval from parents or peers more likely to develop favorable control beliefs, which in turns reinforce vaping intention [21, 37]. These insights suggest that public health strategies should focus on increasing awareness of vaping’s risks, correcting misconceptions, and strengthening parental and school-based prevention efforts. Addressing cognitive factors through targeted interventions could be key to reducing adolescent vaping intention and future initiation.

### Limitation

This systematic review has several limitations. Firstly, studies analyzing young adults aged 20 or older (eight papers) were excluded because they did not focus on participants under 19, the target group of this review. Secondly, despite being based on international literature, the geographic concentration of studies primarily in a few countries (UK, Poland, Spain, Hong Kong and China) and mostly conducted in US, raises concerns regarding the applicability of the results to regions with differing healthcare infrastructures and settings. Thus, future research should consider cultural values, social norms, and policy characteristics to generalize the findings. Thirdly, due to the diverse nature of the studies reviewed, including variations in design, sample populations, and measurement methods such as quantitative synthesis was not feasible. Instead, this review employs a narrative approach, guided by a quality assessment tool, to enable comparisons across different study types. Within the current literature, there is a significant lack of causal evidence, as the majority of information comes from quantitative cross-sectional studies, with only three of longitudinal studies available from the selected article. While cross-sectional studies are valuable for identifying associations, they limit the ability to establish causal relationships between possible predictors and vaping intention due to their study design. Nevertheless, several of these studies, using advanced statistical techniques like regression analysis, enable exploration of potential causal pathways among various factors influencing vaping intentions in adolescents. Aside from that, the concern of publication bias in this systematic review should be highlighted as grey literature was not considered. Additionally, language bias must be addressed because this review only included English-language publication. Despite these limitations, to the author knowledge, this is the first systematic review synthesizing research information on the predictors of vaping intention use among adolescents. Future studies also should aim to conduct meta-analyses to better understand the impact of each predictor.

## Conclusion

This systematic review identified the factors of vaping intention use among adolescents. It provides a comprehensive synthesis of current research on predictors of vaping intention and categorizing findings into five key domains. These key domains includes sociodemographic factors, personality/psychological, social, environmental and tobacco-related cognition. Future longitudinal research should define vaping intention based on the interval duration for future intention to vape, which may vary from soon, next month, next year or even five years. Interventions and programs aimed at increasing effectiveness should address the modifiable risk factors identified in this review. These factors should not be treated in isolation but instead through multifaceted approaches targeting multiple factors simultaneously.

## Data Availability

All data generated or analyzed during this study, included the PRISMA flow chart, quality assessment sheet, and data extraction sheet are available within this manuscript.

## Abbreviations

EC: Electronic cigarettes
NHMS: National Health and Morbidity Survey
NYTS: National Youth Tobacco Survey
US: United States of America
UK: United Kingdom
